# Serological Survey of *Leptospira* Infection in Arabian Horses in Poland

**DOI:** 10.3390/pathogens10060688

**Published:** 2021-06-01

**Authors:** Bernard Wasiński, Katarzyna Paschalis-Trela, Jan Trela, Michał Czopowicz, Jerzy Kita, Monika Żychska, Anna Cywińska, Iwona Markowska-Daniel, Craig Carter, Lucjan Witkowski

**Affiliations:** 1 Department of Microbiology, National Veterinary Research Institute, Al. Partyzantow 57, 24-100 Pulawy, Poland; wasinski@piwet.pulawy.pl; 2 TRELA VETs Referrals, Warsaw, Solec 222, 05-532 Solec, Poland; katarzyna.paschalis@gmail.com (K.P.-T.); trelavets@gmail.com (J.T.); 3 Division of Veterinary Epidemiology and Economics, Institute of Veterinary Medicine, Warsaw University of Life Sciences, Nowoursynowska 159c, 02-776 Warsaw, Poland; mczopowicz@gmail.com (M.C.); Jerzy_kita@sggw.edu.pl (J.K.); monika_zychska@sggw.edu.pl (M.Ż.); iwonamarkowskadaniel@gmail.com (I.M.-D.); lucjan_witkowski@sggw.edu.pl (L.W.); 4 Faculty of Biological and Veterinary Sciences, Nicolaus Copernicus University in Torun, Lwowska 1, 87-100 Torun, Poland; 5 Veterinary Diagnostic Laboratory, Department of Veterinary Science, College of Agriculture, Food & Environment, College of Public Health, University of Kentucky, 1490 Bull Lea Rd, Lexington, KY 40511, USA; craig.carter@uky.edu

**Keywords:** *Leptospira*, seroprevalence, horse, Arabians

## Abstract

Leptospirosis is one of the most common zoonotic infections worldwide, including in most livestock, some companion animals, horses, wildlife, and humans. Epidemiological estimation of its prevalence in all species is difficult due to the variety of clinical presentations and challenges regarding laboratory diagnosis. The purpose of this study was to measure the seroprevalence of leptospiral infection in Arabian horses kept in the largest breeding farms in Poland, representing over 15% of the Polish Arabian horse population. *Leptospira* antibodies were detected by MAT (cut-off 1:100) in 33.2% of serum samples (204 of 615 animals) (CI 95%: 29.6–37.0%), most frequently reacting with the serovar Grippotyphosa, similar to previous reports in populations of randomly selected horses. These results indicated high *Leptospira* seropositivity, thus, although any form of clinical leptospirosis is rare, it may be postulated that the leptospiral exposure is widespread.

## 1. Introduction

Leptospirosis is a ubiquitous infectious and somewhat contagious bacterial zoonotic disease caused by the spirochete *Leptospira* involving many species and serovars. It affects humans, domestic animals, wildlife and even reptiles, posing a worldwide problem for both animal and public health. In horses, the clinical presentation of *Leptospira* infection varies, making a definitive diagnosis often difficult. The majority of equine cases are asymptomatic. Clinical signs are mostly related to the mare’s reproductive tract and kidneys [1,2]. Additionally, *Leptospira* infection can contribute to the development of equine recurrent uveitis (ERU), which appears to be the most important clinical outcome. Although many etiologies are implicated with ERU globally, in continental Europe, infection with *Leptospira* is considered the leading cause. In the US and UK, scientific data also indicate the role of *Leptospira*, which has been isolated from ERU eyes, but the immune mediated process has received more attention [3,4]. Serovars Pomona and Grippotyphosa predominate in equine cases in the US and in Europe, respectively, regardless the clinical presentation.

Due to the fact that chronic intraocular leptospiral infection promotes ERU, any positive serum titer may suggest an increased risk of ERU. The testing of intraocular fluids is the most valuable tool for confirming leptospiral origin of uveitis and also for choosing the proper therapeutic protocol. Detection of pathogenic bacteria or anti-*Leptospira* antibodies in the eye indicates that trans-pars-plana vitrectomy is a treatment of choice for recurrences, whereas in other cases, vitrectomy is less effective in reducing the recurrence rates and protecting the affected eye from further damage [2,3,4,5,6,7,8,9,10].

Serological methods are very useful for leptospirosis screening and sometimes diagnosis (regarding level of titers and/or acute-convalescent samples), however, their efficacy depends on the stage of infection and the immune response of the host. A number of techniques for the detection of antibodies against *Leptospira* serovars has been developed but the microscopic agglutination test (MAT) with live antigens remains the only method acknowledged by the World Organization for Animal Health [11] and is used as the reference method also for screening. 

Almost all epidemiological studies in horses are based on serology with various panels of serovars as test antigens. The reported seroprevalence varied from 1% to over 80% depending on the geographic location, the serovars assessed and the differences in titer cut-off across the studies [12,13,14]. 

In Poland, several studies on leptospirosis in domestic and wild animals have been conducted [15,16], but the data regarding horses are quite limited. To date, reports on the prevalence of *Leptospira* infections in equine species in Poland were published in 1965, 1997 and 2013 [17,18,19], and all of them tested different numbers of serovars and covered populations of various breeds.

Arabians are especially important in Poland, as the Polish Arabian horse breeding program is considered one of the largest and the most successful worldwide. In Polish Arabian populations, ERU is the most common ocular disease, reaching the prevalence of 5.5%, often leading to poor vision and even blindness [20]. Thus, the purpose of this study was to estimate the seroprevalence of *Leptospira* (six serovars) in Arabian horses in Poland. 

## 2. Results 

Among 615 examined samples, 204 were positive at the dilution of 1:100 or more, showing 33.2% (confidence interval-CI 95%: 29.6–37.0%) seroprevalence of *Leptospira* (Table 1). Antibodies reacting with the serogroup Grippotyphosa (MAT is serogroup-specific assay) were found the most frequently (55.9% of positive horses) and the titers ranged from 1:100 to 1:3200. The titers for other investigated serogroups ranged from 1:100 to 1:800 (Table 2). Titers ≥ 1:400 were identified in over 39% of MAT-positive sera reacting with one serovar. There were no significant differences among farms (*p* = 0.78). However, seroprevalence increased with age and differed significantly (*p* < 0.001) between young horses under 4 years (33%) and the horses older than 5 years (50%). Thirty nine of 204 positive sera (19.1%) cross-reacted with two or more serovars (Table 1). In 18 cases (46.2%), reactions with one serovar predominated: Grippotyphosa in 13 cases, Pomona in 2, Sejroe, Tarrasovi and Canicola—one case each.

## 3. Discussion

The results of this study suggest a high exposure rate for *Leptospira* spp. in horses in Poland. The seroprevalence in Arabians (33.2%) is generally consistent with previous studies on mixed populations of healthy horses in northern Poland, indicating 20% [18] and 39.0% [17]. However, a much earlier study indicated seroprevalence as low as approximately 11% in healthy horses but much higher (up to 75%) when related to leptospirosis outbreaks on farms [19], which is consistent with studies from other countries [21,22,23].

There is no standard protocol for the estimation of *Leptospira* seroprevalence. Detection of antibodies directed against leptospires widely varies among investigations because of different MAT titer values considered as “positive” and different antigens used. This study utilized six live antigens for MAT. In the previous reports from Poland, panels of 17, 13 and 8 serovars were analyzed. It is noteworthy that the seroprevalence determined in this study (33.2%) using 6 antigens was higher than in surveys with 8 and 13 antigens (20% and 11%, respectively) and close to the one with 17 antigens (39.0%) [17,18,19].

According to the OIE Terrestrial Manual, titer of 1:100 is interpreted as positive for international trade, but many laboratories do not consider titer to be positive until 1:200 [14] or 1:400 [13]. Sometimes, according to local regulations, other cutoffs are used. For example in Croatia a titer of >400 is considered positive with exception of serovars Bratislava and Australis, when >200 is a positive cutoff [24], while in the latest study from New Zealand the end point titer was recorded at the highest dilution where at least 50% agglutination occurred and it mostly was 1:25 or 1:50 [23]. In this study, the cutoff 1:100 was used to make the data consistent with OIE manual recommendations and to make them comparable with the last study from Poland [17].

It is generally considered that *Leptospira* seroprevalence is lower in temperate climate countries [25] and a seropositivity of 30–45% is expected for tropical regions [14,21]. However, recent data clearly indicate that high seroprevalence is not limited to tropical regions only. Data from Poland previously reported by Arent et al. [17] and confirmed in this study were as high [26] as reported in horses in tropical regions. Additionally, very high seropositivity has been reported in Switzerland and Colorado in the US, where almost 60% and 82% respectively of clinically healthy horses were positive for one or more *Leptospira* antigens [22,27]. On the other hand, in Israel, seroprevalence was low and only individuals out of hundreds investigated horses were seropositive [28]. The risk of *Leptospira* infection may be related to the rainfall during the studies, rodent population increases or decreases or other, sometimes not recognized factors that have to be taken into consideration to interpret and compare reported data properly. This explanation deals also with the differences in seroprevalence of *Leptospira* serogroups in horses among studies from the same country [1,2]. It should be mentioned that the MAT has limitations in the diagnosis of chronic infection in individual animals and in the diagnosis of endemic infections in herds. Infected animals may abort or be renal/genital carriers with MAT titers below the widely accepted minimum significant titer of 1:100 (final dilution) [11].

The Grippotyphosa serogroup has historically been the predominate serovar among horses in central Europe [14]. The results obtained in this study indicate that antibodies against this serogroup were detected in most seropositive horses (55.9%), so agree with previous studies, including the ones conducted in Poland. Zwierz et al. [19] reported in 1965 that the serovar Grippotyphosa was responsible for 30–90% of *Leptospira* infections in horses in Poland. In further studies approximately 10–40% samples were also positive for the this serovar [17,18].

The study from Croatia conducted in 2009–2014 revealed seroprevalence of *Leptospira* spp. from 6.2% to 23.4% (average 13.47%) with the most frequently isolated serogroups being Australis, Pomona and Grippotyphosa [29]. In the earlier Croatian study, 37.2% of horses were positive and the highest seroprevalences were found for serogroups Bratislava, Pomona and Icterohaemorrhagiae [24]. In contrast, the seroprevalence of *Leptospira* spp. among horses in neighboring Serbia was estimated at only 6.3% [30] and the most prevalent serogroups were Grippotyphosa and Icterohaemorrhagiae. 

The seroprevalence in Italy varied from 1.5% [13], through 11.4% [31] to 67.2% [32] and reactions against serovars Bratislava, Canicola, Tarassovi, Copenhageni and Pomona were detected the most frequently, while rarely for Grippotyphosa.

The serological survey carried out in Sweden estimated the prevalence of serovar Grippotyphosa at 0.4% only [25] while serovars Bratislava (16.6%) and Icterohaemorrhagiae (8.3%) were the most prevalent. In Switzerland, serogroups Pyrogenes, Canicola and Australis were the most common among horses [27].

This study covered the representative population involving about 15% of Polish Arabian horses, adding significant data to the epidemiological knowledge base regarding *Leptospira* titers in horses in Poland and Central Europe. The seroprevalence measured in this study was high while clinical evidence of active *Leptospira* infections was not found. Titers confirmed that the exposure is high and does not manifest clinically.

The limitation of this study was the lack of MAT testing against Bratislava serovar. Bratislava is the host adapted serovar found across the globe in horses with the highest seroprevalence. However, it has been shown that at least some of the strains and serovars may not to be pathogenic nor host adapted for horses [33]. It is likely that the use of Bratislava as the antigen would have resulted in even higher seroprevalence than reported here. It also cannot be excluded, that high titers among some of the serum samples reacting in MAT with Grippotyphosa and Canicola serovars may reflect cross-reacting with antibodies against Bratislava.

## 4. Materials and Methods

### 4.1. Selected Population of Arabians

The Polish Arabian Stud Book estimates the entire population of this breed at roughly 4000 animals. Most breeding mares and stallions reside on 3 main Arabian stud farms, all of which are covered in this study. The study population included 425 mares and 190 stallions, a total of 615 horses aged from 6 months to 33 years, representing over 15% of Arabians residing in Poland. Serum samples were collected over 2011–2013 and stored at −20° C until the *Leptospira* titer was tested. Clinical history for the five years prior to sampling was analyzed for each horse [1,2], so clinical records from the years 2006–2013 were checked and revealed no clinical signs suggesting leptospirosis. Samples were taken in the summer season, during routine veterinary procedures, so according to Polish regulations did not require the permission of the Local Ethics Committee.

### 4.2. Analyses 

All sera were tested using the microscopic agglutination test (MAT) (World Organization of Animal Health-OIE, 2019) [11] for six *Leptospira* serovars as follows: Icterohaemorrhagiae, Pomona and Canicola belonging to *Leptospira interrogans*, Sejroe and Tarassovi belonging to *L. borgpetersenii* and Grippotyphosa belonging to *L. kirschneri*. The initial dilution of sera was 1:100, according to the OIE Terrestrial Manual. Sera reacting positively with one or more serovars in the preliminary examination were additionally tested by the twofold dilution series titered to the endpoint defined as dilution of serum that shows 50% agglutination. Farm-level seroprevalences were compared using a chi-square test, *p* < 0.05 was presumed significant.

## 5. Conclusions

It may be postulated that the exposure rate of *Leptospira* spp. in Poland has become higher due to climate change and breeding conditions or increased populations of carriers shedding *Leptospira*. Further, many horses might have been only exposed to this infectious agent, and so only seroconverted without any clinical outcome. Therefore, the occurrence and duration of infections without clinical signs will remain unknown. However, the active onset of infections cannot be excluded and clinical outcomes such as abortion, renal pathology, and septicemia may occur. Thus, the owners, farm managers and the attending veterinarians should be aware of the potential threat of *Leptospira* infection in Arabian horses as a potential zoonotic disease. *Leptospira* infection should be considered as one of the potential pathogens in the diagnostic panels.

## Figures and Tables

**Table 1:** Distribution of equine serum samples reacting in MAT with various serovars of *Leptospira* spp.

Horse Farm	No.of Horses	No. of Positive Horses Seroprevalence (%)	Number of Positive Reactions in 1, 2, 3 and 4 Serotypes								
			Icterohaemorrhagiae	Grippotyphosa	Sejroe	Tarassovi	Pomona	Canicola	In 2 Serotypes	In 3 Serotypes	In 4 Serotypes
A	270	82 (30.4%)	3 (3.7%)	50 (61.0%)	7 (8.5%)	5 (6.1%)	1 (1.2%)	3 (3.7%)	12 (14.6%)	1 (1.2%)	0 (0.0%)
B	292	109 (37.3%)	1 (0.9%)	60 (55.0%)	5 (4.6%)	5 (4.6%)	0 (0.0%)	15 (13.8%)	21 (19.1%)	1 (0.9%)	1 (0.9%)
C	53	13 (24.5%)	0 (0.0%)	4 (30.8%)	0 (0.0%)	5 (38.5%)	1 (7.7%)	0 (0.0%)	3 (23.1%)	0 (0.0%)	0 (0.0%)
Total	615	204 (33.2%)	4 (2.0%)	114 (55.9%)	12 (5.9%)	15 (7.4%)	2 (1.0%)	18 (8.8%)	36 (17.6%)	2 (1.0%)	1 (0.5%)

**Table 2:** Distribution of titers among serum samples reacting in MAT with one serovar.

Serovar	1:100	1:200	1:400	1:800	1:1600	1:3200
Grippotyphosa	18.1%	25.9%	24.5%	17.5%	10.6%	3.4%
Pomona	44.5%	22.2%	11.1%	22.2%		
Tarassovi	78.4%	13.0%	8.6%			
Sejroe	45.5%	40.9%	13.6%			
Canicola	36.8%	42.1%	21.1%			
Icterohaemorrhagiae	53.8%	38.5%	7.7%			

## Data Availability

All data and materials are available in Division of Veterinary Epidemiology and Economics, Institute of Veterinary Medicine, Warsaw University of Life Sciences-SGGW, Nowoursynowska 159c, 02-776 Warsaw, Poland.
